# Interleukin-6 related signaling pathways as the intersection between chronic diseases and sepsis

**DOI:** 10.1186/s10020-025-01089-6

**Published:** 2025-01-31

**Authors:** Jie Yang, Lin Yang, Yanjiao Wang, Lu Huai, Bohan Shi, Di Zhang, Wei Xu, Di Cui

**Affiliations:** Department of Emergency, the People’s Hospital of Liaoning Province, 33 Wenyi Road, Shenhe District, Shenyang, 110016 China

**Keywords:** Sepsis, Chronic diseases, Inflammation, Interleukin-6 related signaling pathways

## Abstract

Sepsis is associated with immune dysregulated and organ dysfunction due to severe infection. Clinicians aim to restore organ function, rather than prevent diseases that are prone to sepsis, resulting in high mortality and a heavy public health burden. Some chronic diseases can induce sepsis through inflammation cascade reaction and Cytokine Storm (CS). Interleukin (IL)-6, the core of CS, and its related signaling pathways have been considered as contributors to sepsis. Therefore, it is important to study the relationship between IL-6 and its related pathways in sepsis-related chronic diseases. This review generalized the mechanism of sepsis-related chronic diseases via IL-6 related pathways with the purpose to take rational management for these diseases. IL-6 related signaling pathways were sought in Kyoto Encyclopedia of Genes and Genomes (KEGG), and retrieved protein-protein interaction in the Search for Interaction Genes tool (STRING). In PubMed and Google Scholar, the studies were searched out, which correlating to IL-6 related pathways and associating with the pathological process of sepsis. Focused on the interactions of sepsis and IL-6 related pathways, some chronic diseases have been studied for association with sepsis, containing insulin resistance, Alcoholic liver disease (ALD), Alzheimer disease (AD), and atherosclerosis. This article summarized the inflammatory mechanisms of IL-6 cross-talked with other mediators of some chronic diseases in vitro, animal models, and human experiments, leading to the activation of pathways and accelerating the progression of sepsis. The clinicians should be highlight to this kind of diseases and more clinical trials are needed to provide more reliable theoretical basis for health policy formulation.

**Supplementary Information:**

The online version contains supplementary material available at 10.1186/s10020-025-01089-6.

## Introduction

Sepsis is a dysregulated immune response associated with host infection, characterized by life-threatening organ dysfunction (Singer et al. 2016; Ye et al. 2020) and evaluated by sepsis related organ failure assessment (SOFA). The main pathophysiology of sepsis is the disruption of the immunological balance. Upregulation both pro-inflammatory and anti-inflammatory mediators leads to a systemic release of cytokines and pathogen-associated molecular patterns, resulting in activation of the complement cascade and multiple organ failure (MOF) (Jarczak et al. 2021). Severe infection, trauma and shock can activate inflammatory cascade reaction and induce sepsis, representation of adult respiratory distress syndrome (ARDS), multiple organ dysfunction syndrome (MODS), and disseminated intravascular coagulation (DIC). The mortality rate rapidly 30%-35%, posing a serious threat and burden to public health (Iba et al. 2021; Vincent et al. 2019). After immune dysregulation in the body, the over-activation of inflammatory mediators can induce Cytokine Storm (CS), inflammatory storms, thrombotic storms, oxidative storms (Alomair et al. 2023). CS are believed to accelerate the development of sepsis (Kang and Kishimoto 2021). Previous studies have suggested that sepsis is often associated with infection, however, chronic diseases such as diabetes, aging, and chronic obstructive pulmonary disease (COPD) can increase the risk of infection and death in sepsis through chronic low-grade inflammation (Chen et al. 2023; Darden et al. 2021; Frydrych et al. 2018).

C-reactive protein (CRP), procalcitonin (PCT), D-dimer and ferritin had been considered as biomarkers in sepsis (Parlato et al. 2018; Vargas-Vargas and Cortes-Rojo 2020). Nowadays, Interleukin (IL)-6 is widely regarding as the center of CS and another biomarker for sepsis (Kang and Kishimoto 2021; Mierzchala-Pasierb and Lipinska-Gediga 2019; Oda et al. 2005). IL-6 belongs to the class of four-helical cytokines with the function of both pro-inflammation and anti‐inflammation (Hunter and Jones 2015). The main cellular source of IL-6 are monocytes, T cells, epithelial cells, tumor cells and tumor-associated fibroblasts (Masjedi et al. 2018; Unver and McAllister 2018), which participate in regulating acute phase reactions, activating T helper (Th) cells, inhibiting T regulatory (Tregs) cells, and differentiating B cells (Scheller et al. 2011). IL-6 binds to its receptor and then activates the transcription factors STAT3 and HIF-1, inducing signaling pathways activation. CS and IL-6 related signaling pathways transduction are considered as crucial factors involved in sepsis, such as upregulation of IL-6/JAK/STAT3 signaling promotes the progression of sepsis (An et al. 2023), involvement of IL-6 signaling pathway in the pathogenesis of MOF (Kang and Kishimoto 2021) and other hyperinflammatory diseases (Cron et al. 2023). Targeting IL-6 and/or IL-6 receptor has been seen as a clinical immunotherapy strategy for CS (Kang and Kishimoto 2021; Xu et al. 2020c). IL-6 cross-talked with various types of immune factors can activate other pathways, containing TLR signaling pathway (Kumar 2020), ERK1/2 and p38 MAPK signaling pathway (Riedemann et al. 2004), NF-κB signaling pathway (Bauernfeind et al. 2009), and JAK-STAT signaling pathway (Lu et al. 2014).

CRP, PCT, D-dimer, and ferritin can reflect the severity of sepsis. Furthermore, sepsis is related to infection and/or significant inflammation, however, the unreasonable management of chronic diseases was prone to induce sepsis through the chronic low-grade inflammation. In the previously studies, most clinicians paid more attention to infection and trauma instead of management for chronic diseases. Therefore, this review centered on IL-6 related signaling pathways and the biomarkers to analyze sepsis and sepsis-associated chronic diseases with the aim to guide clinicians in the effective diagnosis and treatment, prediction outcomes, and assessment risk for these diseases. Moreover, this study summarizes previous animal and human experiments, providing a theoretical basis for further clinical trials and facilitating the formulation of public health policies.

## Seek out IL-6 related signaling pathways and sepsis-associated chronic diseases

We sought for IL-6 related signaling pathways in the Kyoto Encyclopedia of Genes and Genomes (KEGG), and got a total of 51 pathways. Firstly, selected the directly upstream participants, downstream products, and intermediate products centering around IL-6 (as shown in Table 1). Secondly, retrieved the differentially expressed genes (DEGs) of the biomarkers, including the upstream participants and the downstream products in the National Library of the United States (National Center for Biotechnology Information), and screened the relevant DEGs. The directly related DEGs was shown in Table 2. Thirdly, the protein-protein interactions for these related DEGs, CRP, PCT, D-dimer and ferritin were observed via the tool of Search Tool for the Retrieval Interacting Genes (STRING), and the high-frequency IL-6-related pathways and the relevant chronic diseases (Table 3) were screened out using Cytoscape software and the Cytohub application. We searched PubMed and Google Scholar for articles published in English relating to the selected pathways and chronic diseases (Table 3), and summarized these interactions. The screened literatures must meet the conditions of signaling pathways that can induce sepsis and chronic disease-related inflammatory factors that can activate pathways associating to sepsis.

**Table 1:** IL-6 related signaling pathways

Type	No.	KEGG map	Signaling pathway	Target spot	Upstream	Downstream	Identity
Cytokine Signaling Pathways	1	map04066	HIF-1 signaling pathway	IL-6	-	STAT3	Participant
	2	map04630	JAK-STAT signaling pathway	IL-6 family	-	JAK1/2, TYK2, STAT1/3/6	Participant
	3	map04657	IL-17 signaling pathway	IL-6	NF-κb, IKBA, MAPKs, AP1, TRAF6, C/EBP-b	-	Product
	4	map04659	Th17 cell differentiation	IL-6	-	JAK1/2, STAT3	Intermediate products
	5	map04668	TNF signaling pathway	IL-6	AP-1, C/EBP-b, CREB	-	Product
	6	map04933	AGE-RAGE signaling pathway in diabetic complications	IL-6	ERK1/2, P38, AP-1, JNK/Akt, NF-κb	-	Product
	7	map05022	Pathways of neurodegeneration - multiple diseases	IL-6	NF-κb	Reduction in LTP, AGE-RAGE signaling pathway in diabetic complications	Intermediate products
	8	map05200	Pathways in cancer	IL-6	C/EBP-a target genens	Block of differentiation	Intermediate products
	9	map04068	FoxO signaling pathway	IL-6	-	STAT3	Participant
	10	map04151	PI3K-Akt signaling pathway	IL-6	-	-	Participant
	11	map04620	Toll-like receptor signaling pathway	IL-6	AP-1, NF-κb, IKB, IRF5	-	Product
	12	map04621	NOD-like receptor signaling pathway	IL-6	AP-1, NF-κb, IKB	-	Product
	13	map04623	Cytosolic DNA-sensing pathway	IL-6	NF-κb, IKB	-	Product
	14	map04625	C-type lectin receptor signaling pathway	IL-6	p65/p50, BCL-3/p50	Th1/Th17, Th2	Product
Chronic Diseases	15	map04672	Intestinal immune network for IgA production	IL-6	DC, Lamina propria stoma cell	-	Product
	16	map04931	Insulin resistance	IL-6	Obesity, NF-κb, IKBA,	STAT3, SOCS3	Participant
	17	map04932	Non-alcoholic fatty liver disease	IL-6	NF-κb	SOCS3	Participant, Product
	18	map04936	Alcoholic liver disease	IL-6	NF-κb, IKBA	-	Product
	19	map05010	Alzheimer disease	IL-6	ERK1/2, ROS, NF-κb	-	Intermediate products
	20	map05321	Inflammatory bowel disease	IL-6	Macrophage, APC	-	Participant, Product
	21	map05323	Rheumatoid arthritis	IL-6	DC, Synovial macrophage, Synovial fibroblast	-	Participant, Product
	22	map05332	Graft-versus-host disease	IL-6	Host tissues	Host APC	Participant
	23	map05410	Hypertrophic cardiomyopathy	IL-6	Left ventricular remodeling	Left ventricular hypertrophy	Intermediate products
	24	map05417	Lipid and atherosclerosis	IL-6	NF-κb, IKBa	-	Product
Virus Infection	25	map05020	Prion disease	IL-6	-	TNF-α signaling pathway	Participant
	26	map05161	Hepatitis B	IL-6	NF-κb, IKB	-	Product
	27	map05162	Measles	IL-6	AP-1, NF-κb, IKB	-	Product
	28	map05163	Human cytomegalovirus infection	IL-6	NF-κb, IKB	-	Product
	29	map05164	Influenza A	IL-6	NF-κb, IKB	-	Product
	30	map05166	Human T-cell leukemia virus 1 infection	IL-6	NF-κb, IKB	-	Product
	31	map05167	Kaposi sarcoma-associated herpesvirus infection	IL-6	HIF-a, NF-κb, AP-1	-	Product
	32	map05168	Herpes simplex virus 1 infection	IL-6	NF-κb, IKB	-	Product
	33	map05169	Epstein-Barr virus infection	IL-6	NF-κb, IKB	-	Product
	34	map05171	Coronavirus disease - COVID-19	IL-6	NF-κb, IKB	-	Participant, Product
	35	map04061	Viral protein interaction with cytokine and cytokine receptor	IL-6	-	-	Participant
Bacterial Infection	36	map05130	Pathogenic Escherichia coli infection	IL-6	AP-1	-	Product
	37	map05132	Salmonella infection	IL-6	AP-1, p50, p65	-	Product
	38	map05133	Pertussis	IL-6	NF-κb, IRF3, AP-1	-	Product
	39	map05134	Legionellosis	IL-6	MyD88, Toll-like receptor signaling pathway	-	Product
	40	map05135	Yersinia infection	IL-6	NF-κb, IKBA, ERK/P38/JNK, AP-1	-	Product
	41	map05152	Tuberculosis	IL-6	NF-κb	-	Product
Infectious Diseases	42	map05142	Chagas disease	IL-6	JNK/ERK1/2, P38, AP-1	-	Product
	43	map05143	African trypanosomiasis	IL-6	Macrophage	-	Product
	44	map05144	Malaria	IL-6	MyD88	-	Product
	45	map05146	Amoebiasis	IL-6	NF-κb	N infiltration	Product
Others	46	map05202	Transcriptional misregulation in cancer	IL-6	C/EBP-b	proliferation and invasion	Product
	47	map01521	EGFR tyrosine kinase inhibitor resistance	IL-6	-	Epithelial mesenchymal transition (EMT)	Participant
	48	map01523	Antifolate resistance	IL-6	NF-κb	-	Product
	49	map04060	Cytokine-cytokine receptor interaction	IL-6	-	-	Participant
	50	map04218	Cellular senescence	IL-6	NF-κb, ZPF36L, Calpain, IL1a	Paracrine senescence	Intermediate products
	51	map04640	Hematopoietic cell lineage	IL-6	Hematopoietic cell	-	-

**Table 2:** The interactions of the directly related DEGs

DEGs	Gene Symbol	CRP	PCT	D-dimer
MAPKs	*MAPK14*,* MAPK13*,* MAPK12*,* MAPK11*,* MAPK1*,* MAPK6*,* MAPK3*,* MAPK7*,* MAPK4*	**+**	**-**	**+**
CREB	*CREB1*	**-**	**-**	**-**
IRF5	*IRF5*	**-**	**-**	**-**
AP-2	*AP2A2*,* AP2S1*,* AP2M1*,* AP2B1*,* AP2A1*	**-**	**-**	**-**
HIF-a	*-*	**-**	**-**	**-**
IRF3	*IRF3*	**-**	**-**	**-**
JNK	*MAPK8*,* MAPK8IP1*,* MAPK8IP2*,* MAPK8IP3*	**-**	**-**	**-**
C/EBP-b	*CEBPB*	**-**	**-**	**-**
ZPF36L	*-*	**-**	**-**	**-**
BCL-3	*BCL-3*	**-**	**-**	**-**
p65	*SYT1*	**-**	**-**	**-**
Calpain	*CAPN5*,* CAPN6*,* CAPN7*,* CAPN8*,* CAPN9*,* CAPN10*,* CAPN11*,* CAPN12*,* CAPN13*,* CAPN14*,* CAPN15*,* CAPNS1*,* CAPNS2 CAPN3*,,	**-**	**-**	**-**
TRAF6	*TRAF6*	**-**	**-**	**-**
IL1a	*IL1R1*			
Akt	*AKT1*,* AKT2*,* AKT3*	**+**	**+**	**-**
p50	*NFKB1*	**-**	**-**	**-**
AP-1	*AP1B1*,* AP1G1*,* AP1S1*,* AP1S2*,* AP1S3*,* AP1M2*	**-**	**-**	**-**
NF-κb	*NFKB1*	**-**	**-**	**-**
P38	*MAPK14*,* MAPK13*,* MAPK12*,* MAPK11*	**-**	**-**	**-**
IKBA	*NFKBIA*	**+**	**-**	**-**
ERK1	*MAPK3*,* MAP2K2*	**+**	**-**	**+**
IKB	*IKB*	**-**	**-**	**-**
MyD88	*MyD88*	**+**	**-**	**-**
ERK2	*MAPK1*	***-***	**-**	**+**
TYK2	*TYK2*	**-**	**-**	**-**
STAT1	*STAT1*	**-**	**-**	**-**
STAT6	*STAT6*	**-**	**-**	**-**
JAK1	*JAK1*	**-**	**-**	**-**
JAK2	*JAK2*	**+**	**-**	**-**
Th1	*-*	**-**	**-**	**-**
Th17	*-*	**-**	**-**	**-**
Th2	*-*	**-**	**-**	**-**
STAT3	*STAT3*	**+**	**-**	**-**
SOCS3	*SOCS3*	**-**	**-**	**-**
SOCS4	*SOCS4*	**-**	**-**	**-**
IL-6	*IL-6*	**+**	**+**	**+**
IL-6R	*IL-6R*	**+**	**-**	**-**

The plus sign (+) indicates that the DEGs are directly related to CRP, PCT, and D-dimer. Previously studies have shown that although ferritin was mainly involved in autoimmune diseases, it mainly occurred in sepsis-associated kidney injury (McCullough and Bolisetty 2020; Zandman-Goddard and Shoenfeld 2007, 2008). The DEGs of Ferritin only contacted to its two subunits instead of interacting with the other DEGs.

**Table 3:** The related signaling pathways and sepsis-associated chronic diseases

KEGG map	Signaling pathway
map04066	HIF-1 signaling pathway
map04630	JAK-STAT signaling pathway
map04657	IL-17 signaling pathway
map04659	Th17 cell differentiation
map04933	AGE-RAGE signaling pathway in diabetic complications
map04068	FoxO signaling pathway
map04931	Insulin resistance
map04936	Alcoholic liver disease
map05010	Alzheimer disease
map05417	Lipid and atherosclerosis

## The interactions between IL-6 related signaling pathways and sepsis

CRP and PCT are the pro-inflammatory biomarkers for evaluating the severity of sepsis, while D-dimers is considered a thrombotic biomarker for accessing the progression of CS (Al-Kuraishy et al. 2023; Parlato et al. 2018). In the previous studies, IL-6 and its receptors were considered as the key nodes in immunotherapies of sepsis (Boomer et al. 2014; Xu et al. 2020c). The DEGs of CRP, PCT and D-dimer interacted with the upstream participants and/or downstream products of IL-6 and its related signaling pathways. As a result, immune cascade reaction expanded continuously, leading to sepsis and/or septic shock ultimately. Therefore, sepsis is associated to the activation of IL-6 related signaling pathways.

### The IL-6 related signaling pathways and sepsis

IL-6 induced other inflammatory mediators/transcription factors, resulting in activation of the inflammatory pathways and promoting the occurrence of CS. Other studies have found that CS can cause stress reaction in the body, leading to strengthening of sympathy activation and triggering stronger inflammatory storms, through inflammatory mediators cross-talking with each other in hypercytokinemia (Al-Kuraishy et al. 2021). Previous study shown that IL-6 and TNF-α were at the beginning of inflammatory cascade, inducing capillary leak and leading circulatory collapse (Pearce et al. 2020). However, other studies shown the anti-inflammatory function of IL-6. IL-6 induced Th cells producing IL-17 to secrete IL-10 (Stumhofer et al. 2007). IL-10 is known as an inhibitory factor, with effects on the expression of inflammatory cytokines, such as TNF-α, IL-6, IL-1, IL-8, IL-12, and IL-18. In T cells, STAT3 is essential for IL-6-induced IL-10 production (Akdis and Blaser 2001; Mosser and Zhang 2008). Moreover, STAT3 can recruit to IL-10 receptor1 upon IL-10 binding, driving the expression of anti-inflammatory mediators (Donnelly et al. 2004). Previous study shown that hyperinflammatory innate immune response initially was mainly an overactive IL-1/IL-6 response (van de Veerdonk and Netea 2020). Other studies have shown that the IL-1/IL-6 axis was involved in regulating inflammatory responses (Lust et al. 2016). IL-6 can be secreted by IL-1 stimulation monocytes and/or macrophages. Furthermore, IL-1 antagonist was seen as a possible mechanism to limit IL-6 generation in septic inflammation (Privratsky et al. 2023). Similar studies shown, Anakinra blocked the IL-1 receptor via inhibiting the effects of IL-1α released from dead epithelial cells, as well as IL-1β produced by immune cells. IL-1-induced IL-6 will also be inhibited (Shakoory et al. 2016; van de Veerdonk and Netea 2020). These inflammatory pathways activated by IL-6 cross-talked with inflammatory factors are considered as IL-6 related pathways. We have summarized the interactions of IL-6 related signaling pathways (Table 3) and sepsis, as shown below.

#### HIF-1 signaling pathway and JAK-STAT signaling pathway

STAT3 is located at downstream of the IL-6 pathway and can induce HIF-1 to activate the HIF-1 pathway (Semenza 2010). HIF-1α is seen as an earlier participant in the inflammatory cascade of septic mitochondrial dysfunction (Regueira et al. 2009). Previous studies have shown, monocytes generated immunosuppression through HIF-1α signaling pathway in the later phase of sepsis (Li et al. 2^020^). In mice model, HIF-1α signaling pathway was related to sepsis-induced lung injury, ARDS and encephalopathy (Ding et al. 2023; Zhao et al. 2021, 2022). Some Chinese medicine studies shown that HIF-1α signaling pathway improved the prognosis of sepsis by inhibiting aerobic glycolysis, regulating cell apoptosis, and responding to inflammation and oxidative stress (Ding et al. 2023; Lu et al. 2020). STAT3 is a crucial node in JAK-STAT signaling pathway, and involved in the early stage of septic inflammatory response (Yuan et al. 2023). Other studies have shown that, in the context of SARS-CoV-2 infection, the binding of IL-6 to its receptor can trigger other inflammatory pathways though STAT3, leading to excessive inflammatory responses and CS (Alomair et al. 2023). IL-6 induced muscle atrophy of mice (Zanders et al. 2022) and acute lung injury (ALI) in sepsis through JAK-STAT signaling pathway (Chang et al. 2019; Wang et al. 2022a; Zhang et al., 2023b), and IL-6 was the favorable target for sepsis inflammation (Pearce et al. 2020). In the study of sepsis-related myocardial injury, JAK-STAT signaling pathway was identified in inflammatory response and apoptosis of myocardial cell (Jin et al. 2020; Tarasiuk et al. 2020). Meanwhile, the JAK2-STAT3 signaling pathway was involved in inflammation and cell apoptosis in septic acute kidney injury (AKI) (Zhu et al. 2020). However, other studies have shown that in addition to IL-6, IL-1 and TNF can also activate the JAK-STAT pathway. For instance, IL-1-induced signaling cascade can activate JAK-STAT pathway to promote inflammation (Biffi et al. 2019). TNF-α induced the highest levels of IL-6 release from JAK-STAT3 pathways (Matsumoto et al. 2018).

#### IL-17 signaling pathway and Th17 cell differentiation

JAK-STAT3 can promote the differentiation of CD4 + T cells into Th17 cells. The activated Th17 cells assisted the expression of IL-17 (Shibabaw 2020). Another study found that TNF-α acted as an upstream mediator of Th17 cells which produced IL-17 (Wolk et al. 2020). IL-17 binds to its receptor and activates the IL-17 pathway, which can lead to the expression of pro-inflammatory cytokines and chemokines (Brembilla et al. 2018). In the study of septic visceral organ injury, IL-17 and IL-17 signaling pathway have been confirmed to be involved in ALI and endotoxic shock (Alnfakh et al. 2022; Giangola et al. 2013; Kwon et al. 2019; Wang et al. 2022c; Zhang et al. 2023a). In the studies of animal models in sepsis, Th17 cell differentiation was induced by macrophages (Qin et al. 2012), involved in inflammatory response (Zou et al. 2020), and associated with AKI (Luo et al. 2023). However, Th17 cells are not essential for the development of LPS-induced endotoxin shock (Shimura et al. 2014).

#### AGE-RAGE signaling pathway and FoxO signaling pathway

AGEs are the products of non-enzymatic glycation and oxidation of proteins and lipids. AGEs interaction with RAGE can activate ERK pathways, which are involved in many human inflammatory diseases (Linke et al. 2014). The variation of IL-6 levels is positively correlated with the expression of ERK (Vetuschi et al. 2020). In addition, KEGG pathway analysis showed that human cytomegalovirus active viremia was associated with AGE-RAGE signaling pathway in diabetes complications (Parhizgari et al. 2023). FoxO family plays a role in basic cellular processes such as immune regulation, metabolism and apoptosis (Wei et al. 2021). FoxO has been considered as a STAT3 target gene (Graves and Milovanova 2019). Inhibition of FoxO1 can reduce the expression of IL-6 (Hutchins et al. 2013). In FoxO signaling pathway, acetylated forkhead box O1 was associated with sepsis induced AKI in mice (Li et al. 2021), while Akt/FoxO was related to muscle atrophy and insulin resistance in rats (Crossland et al. 2008).

**Fig. 1:**
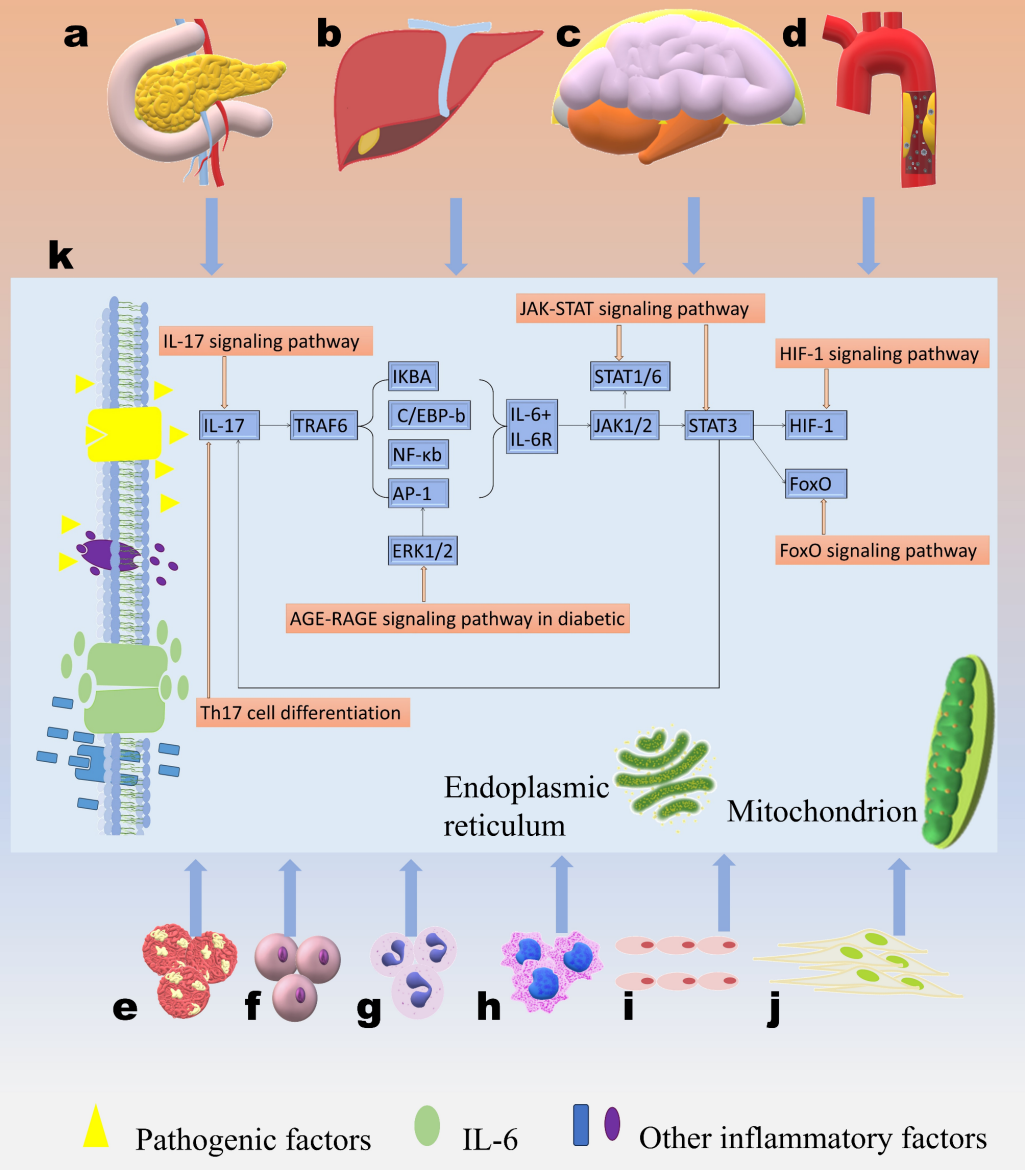
The mechanism map of sepsis-related chronic diseases via IL-6 related signaling pathways. (a-d) These diseases, containing insulin resistance (**a**), Alcoholic liver disease (ALD) (**b**), Alzheimer disease (AD) (**c**), and Lipid and atherosclerosis (**d**). (**e-j**) The cellular source of IL-6, containing tumor cells (**e**), T cells (**f**), monocytes (**g**), macrophages (**h**), epithelial cells (**i**), and tumor-associated fibroblasts (**j**). (**k**) The inner of these immunocytes. IL-6 binds to its receptor on the surface of these cells, and combines transcription factors (the nodes of signaling pathways), containing IL-17 (node of IL-17/Th17 axis pathways), IKBA, C/EBP-b, NF-κb, AP-1and ERK1/2 (nodes of AGE-RAGE pathway in diabetic); at downstream includes STAT1/3/6 and JAK1/2 (nodes of JAK-STAT pathway), HIF-1 (node of HIF-1 pathway), and FoxO (node of FoxO pathway). STAT3, the classic transcription factor in the JAK-STAT pathway, can activate upon binding of IL-6 to its receptor. JAK-STAT3 can promote expansion of CD4 + T cells to Th17 cells. The activated Th-17 cells produce inflammatory cytokines such as IL-17. IL-17 binds to its receptor, transduces signals and recruits TRAF6, leading to phosphorylation and proteasomal degradation of IκB, ultimately activating inflammatory cytokines and chemokines that encode genes targeted by NFκB. The nodes are cross-talked with others, subsequently, these related signaling pathways were activated respectively. However, other inflammatory factors were participated in the inflammatory cascade, such as pro-inflammatory cytokines TNF and IL-1. TNF and IL-1 were seen as the earlier mediators in cascade, while IL-6 was seen as the center of inflammation and these pathways were considered as IL-6 related signaling pathways. Under the influence of pathogenic factors, inflammatory mediators were activated, then the inflammatory cascade gradually intensified via IL-6 related pathways and induced CS, resulting in sepsis. The function of crucial organs was destroyed by the disruption of the immunological balance via these signaling pathways. As a result, ARDS, MODS, and DIC were happened. Finaly, sepsis developed into septic shock

In our study, there are many IL-6 related signaling pathways that can induce sepsis, and the activation of these inflammatory signaling pathways is related to these factors cross-talking, therefore, our team has drawn a mechanism map (Fig. 1).

### The interactions of chronic diseases, sepsis and IL-6 related signaling pathways

The action of IL-6 related signaling pathways can make effects on glucolipid metabolism, as a result internal environment was changed and chronic diseases could be exposed in inflammatory conditions. For example, obesity is one of the results of abnormal glucose and lipid metabolism. It increases the risk of insulin resistance, steatohepatitis, AD, cardiovascular disease, and other comorbidities. In these tissue, inflammatory processes play a crucial role in exacerbating chronic inflammation and activate Tregs, macrophages and Th1 cells, even inflammatory signaling pathways (Shantaram et al. 2024). Ultimately, inflammatory factors are released and immune cascade reaction occurs, leading to sepsis. Next, we will generalize the four sepsis-related chronic diseases and six IL-6 related signaling pathways in Table 3.

#### Insulin resistance

Insulin resistance is a crucial kind of metabolic syndrome and a precursor of type 2 diabetes mellitus (T2DM) (Yang et al. 2023). Chronic low-level inflammation is the main cause for insulin resistance (Sorski and Gidron 2023). Previous study shown that IL-1β contributed to the pathogenesis of obesity-induced insulin resistance (Ballak et al. 2015), and associated with HIF-1 production (Torretta et al. 2020). Patients with sepsis have elevated insulin levels but decreased insulin sensitivity (Rivas and Nugent 2021). The impact of related signaling pathways on glucose metabolism is as follows. HIF signal transduction was reduced along with the reduction in glycolytic enzymes of human with T2DM, with the special node HIF1-β (Gunton et al. 2005). HIF1-α knockout in β cells also impaired insulin secretion of human and mice (Cheng et al. 2010). JAK-STAT pathway is dysregulated in the context of obesity and metabolic disease, moreover, JAK-STAT related proteins was involved in the insulin cascade response and considered as the treatment of obesity and diabetes (Collotta et al. 2023; Dodington et al. 2018). JAK2 joined in phosphorylate insulin receptor substrate and glycolipid metabolism (Ge et al. 2008). IL-17 A, a core target in the IL-17 signaling pathway, took part in glucose metabolism with the function of preventing diabetic nephropathy (Mohamed et al. 2016). In the previous research, the imbalance of the Th17/Treg cell ratio and STAT3 protein were play an important role of obesity with hyperinsulinemia (Liu et al. 2021). In mice model, Th17 cells were joined in mediating insulin resistance of obesity (Meissburger et al. 2011). In the study for the function of dendritic cells shown that Th17 cell differentiation was associated with obesity-related insulin resistance (Bertola et al. 2012). In the mice model and case-control study shown that the accumulation of AGEs in the extracellular matrix may affect the metabolism of insulin sensitivity (Csongova et al. 2022; Monden et al. 2013). The AGE-RAGE axis in adipose tissue also appeared to play a key role in the development of obesity-related insulin resistance (Gutowska et al. 2023). FoxO1, a member of the “Forkhead box” transcription factor family, can promote the reduction of circulating glucose by binding to insulin (Sin et al. 2015). Therefore, if a patient with insulin resistance, sepsis is likely to occur after the pathogenic factors activate the aforementioned signaling pathways. The mechanism is shown in Fig. 1 for these related nodes and pathways in the inner of inflammation cells.

#### ALD

ALD is caused by long-term or large volumes of alcohol intake, which leads to liver injury, fatty liver, inflammation, fibrosis and cirrhosis, and is accompanied by metabolic disorder of glucose and lipid (Seitz et al. 2018). Inflammation acts as a key factor driving ALD progression, containing infiltration of neutrophils and macrophages, as well as activation of Kupffer cells and other kinds of immune cells (Gao et al. 2019). Sepsis is one of the most common complications and causes of death in patients. Impaired innate and adaptive immunity can lead to increase susceptibility to infection, in ALD patients (Vassallo et al. 2021). Other studies shown that HIF-1α and HIF-2α were involved in the pathogenesis of ALD (Ju et al. 2016; Wang et al. 2011). In mice model, the activation of Akt/HIF-1α pathway was promoted in the inflammatory response of alcohol induced fatty liver disease (Wang et al. 2021). Moreover, hepatocytes were more susceptible to the inhibitory effects of ethanol through the JAK-STAT pathway (Chen et al. 2001).Other studies shown that ethanol significantly inhibited IL-6-induced STAT3 phosphorylation through the JAK-STAT pathway (Chen et al. 1999). IL-17 A was a common mediator of excessive alcohol consumption and alcohol induced liver/brain injury, regulating alcohol induced hepatic steatosis, inflammation, and fibrosis in mice (Ma et al. 2020; Xu et al. 2020a). Furthermore, IL-17 signaling pathway was involved in the development of ALD, anti-IL-17 antibody can improve hepatic steatosis (Shi et al. 2013). In the study of the interaction of fibrosis-associated macrophages, natural killer cell and mucosal-associated invariant T cells revealed that IL-17 signaling pathway and Th1/Th2/Th17 cell differentiation were considered to be regulated in the intra-fibrotic activity of several pro-fibro genic pathways (Ren et al. 2023). The traditional Chinese medicine, ginsenoside F2, had alleviated alcoholic liver injury by reducing IL-17 expression and Th17 cells in mice (Kim et al. 2020). However, the AGE-RAGE signaling pathway in diabetic complications was the most common pathway in non-alcoholic fatty liver disease through bioinformatic analysis, for example AKT1, IL-6, and STAT3 were considered as the key target nodes (Hu et al. 2020; Mahmoudi et al. 2022). In FoxO pathway, FoxO1 and SIRT1 were took crucial role in the pathogenesis of Alcoholic hepatitis, a severe form of ALD (Yao et al. 2019). In the FoxO3/Bim signaling pathway, FoxO3 made an effect on Bim decreasing, as a result the apoptosis of hepatocytes was decreased in primary biliary cirrhosis in mice (Kopycinska et al. 2013). Therefore, the activation of the above pathways is highly susceptible to sepsis, and the related mechanism is shown in Fig. 1.

#### AD

AD is the most common type of dementia, with the character of white matter loss and myelin degeneration due to death of oligodendrocytes occurring in the early phase (Perrone et al. 2012). In the study of AD, endotoxin contributes to neurodegeneration and induces microglial activation, initiation and/or tolerance, memory deficits, and loss of brain synapses and neurons (Brown 2019). Therefore, AD is more likely to occur after sepsis and its complications. The relevant pathways and their main nodes are as follows. The expression of HIF-1 inhibited progression in patients and enhanced neuroprotective compensation pathways for many physiological processes in brain (Crapper McLachlan et al. 1991; Weinreb et al. 2012). For example, the transcription of HIF-1α dependent genes relates to the expression of vascular endothelial growth factor and tyrosine hydroxylase in primary cortical cells of rats (Avramovich-Tirosh et al. 2010). In some medical experiments, the level of protein of HIF-1α was elevated with AD-mice. Deferoxamine can inhibit the expression of HIF-1α, it will be seen as a potential treatment for AD (Fine et al. 2012; Gedam et al. 2023; Khuu et al. 2021). In AD, reactive astrocytes regulated synaptic signaling and responded to central nervous system damage through JAK-STAT signaling pathway (Qin et al. 2023). Other medical research shown that Abeta peptide caused neuronal death through the JAK-STAT signaling pathway, leading to AD (Buckingham et al. 2009). SOCS, the downstream of JAK-STAT pathway, can facilitate the manipulation of inflammatory response, leading to degenerative brain diseases (Woo et al. 2015). Similar effects were found in microglia (Choubey 2019). In mice model, the impaired Treg function was associated with AD pathology, and the recovery of Treg dysfunction reduced hippocampal inflammation (Abdelmoaty et al. 2023). The elevation of IL-17 A levels was related to the activation of astrocytes in the hippocampus and the appearance of AD pathological features (Tian et al. 2015). In female AD patients, decreasing communication between excitatory neurons and microglia was related to IL-17 pathway (Hou et al. 2023). In various neurological diseases, the frequency of Th17 infiltration in the blood-brain barrier was significantly upregulated (Kubick et al. 2023). In these diseases, Th17 cell differentiated to a highly pro-inflammatory phenotypes, broke through the blood-brain barrier, recruited more inflammatory cells to participate in neuroinflammation, and ultimately lead to neurodegeneration (Dai et al. 2022). In AD patients, the proportion of Th17 cells in peripheral blood was higher than healthy controls, and the concentration of IL-17 in serum was also higher (Milovanovic et al. 2020). In the study of impaired glucose and lipid metabolism, FoxO signaling and insulin signaling shared a common pathological mechanism for AD and diabetes (Pardeshi et al. 2017). In addition, FoxO signaling pathway was related to aging (Guo et al. 2017). Therefore, sepsis is likely to induce or exacerbate AD through the above pathways, and the related mechanism is shown in Fig. 1.

#### Lipid and atherosclerosis

Atherosclerosis is a common ageing disease characterized by chronic inflammatory process resulting from an imbalance in lipid metabolism and vascular function (Figueiredo et al. 2023). Inflammation plays a central role in atherosclerosis and its complication (Bezsonov et al. 2021). Lipid disorder in septic survivors may be associated with atherosclerosis (Felici et al. 2021). Previous study shown, IL-6 can trigger inflammatory cells in the atherosclerotic plaques to secrete inflammatory cytokines and induce myocardial ischemia. Under the condition of hypercytokinemia caused by virus infection, myocardial injury is more likely to occur without acute coronary atherothrombosis (Alsaidan et al. 2023). Animal models showed that blocking HIF pathway had beneficial effects on atherosclerosis. It means the target gene of HIF transcription was related to affect cholesterol uptake, migration, and inflammation (Liu et al. 2016; Norda and Papadantonaki 2023). Similar studies in cardiovascular medicine shown that atherosclerosis angiogenesis was mediated by HIF-1α signaling pathway (Chen et al. 2018). Moreover, macrophage was participated in HIF-1α signaling pathway to enhance the effects of atherosclerosis. The expression of HIF-1α was up-regulation with atherosclerosis. However, HIF-1α inhibition abrogated the proangiogenic effect of oxidized low-density lipoprotein (Aarup et al. 2016; Hutter et al. 2013; Wang et al. 2022b). In mice model, JAK-STAT signaling pathway was promoted to atherosclerotic inflammation with increasing the phosphorylation of JAK2, STAT1 and STAT3 (Fu et al. 2022). The activation of JAK-STAT pathway can inhibit cholesterol efflux and promote inflammatory cytokine release and cell apoptosis in patients (Li et al. 2018; Mo et al. 2011). Other studies shown that inhibition of the protein expression levels of SOCS3, and phosphorylation of p-JAK1 and p-STAT1in JAK-STAT axis was proved to be a new method for clinical treatment of atherosclerosis (Hashimoto et al. 2020; Yang et al. 2020; Yue et al. 2023). In IL-17 signaling pathway, IL-17 A is involved in lipid metabolism, proatherogenic and also plays an important role in diet-induced atherosclerotic lesion development (Chen et al. 2010; Yu et al. 2014). IL-17 can increase apoptosis of endothelial cells which induced by lipopolysaccharide (Xu et al. 2020b). Smooth muscle cell-derived IL-17 C promoted atherosclerosis through recruiting Th17 cells into atherosclerotic lesions (Butcher et al. 2016). Other study has shown that the imbalance of Th17/Treg cell exacerbated the development of atherosclerosis (Tsilingiri et al. 2019). In addition, the reduction of inflammatory factors can inhibit the differentiation of Th17 cells, and reduce the production of IL-17 A in the atherosclerosis microenvironment (Zhou et al. 2022). In the study of AGE-RAGE signaling pathway in diabetes complications, AGEs-their receptor RAGE evoked vascular inflammation, and was involved in the pathogenesis of accelerated atherosclerosis of diabetes (Yamagishi et al. 2008). In the study of FoxO signaling pathway, FoxO1, a crucial regulatory factor of cellular metabolism in several tissues, was involved in cardiac regulation of glucose and lipid metabolic pathways (Menghini et al. 2020). Furthermore, FoxO1/3 was considered to take part in vascular calcification of atherosclerosis (Deng et al. 2015). Therefore, activation of these pathways is more likely to lead to Lipid related atherosclerosis after sepsis, and the related mechanism is shown in Fig. 1.

## Conclusions

IL-6 was the core of CS in sepsis. IL-6 related signaling pathways are equivalent to a network of signaling pathways via IL-6 cross-talking with nodes. Therefore, the inflammatory cascade reaction gradually strengthens, leading to CS-induced sepsis. By activating the IL-6 related signaling pathway, some chronic diseases are prone to sepsis via stimulated by chronic low-grade inflammation, meanwhile, sepsis can exacerbate these chronic diseases and lead to MOF. These chronic diseases contained insulin resistance, ALD, AD and atherosclerosis. As mentioned above, TNF can activate STAT3, HIF-1a, Th17, and IL-6, while IL-1 can induce insulin resistance, HIF-1 production, and JAK-STAT signals. It means that they are not exclusive IL-6 pathways. Therefore, we considered these factors as the earlier mediators in the inflammatory cascade. So, take effective management to blocked over-activation of IL-6 related signaling pathways in these chronic diseases may be seen as the better method for prevention and treatment for sepsis. Our review focused on CS, IL-6 related signaling pathways, and sepsis-related chronic diseases. However, most studies that reference the IL-6 activation pathways have been evaluated in vitro and animal models, therefore, more in vivo and clinical trials are needed to increase data credibility and provide more reliable theoretical basis for health policy formulation. In the studies of sepsis, CS is just one branch of the pathogenesis of sepsis. Further research will consider to explore the interaction of mediators for inflammatory storms, thrombotic storms, oxidative storms, and the overlapping storms.

## Electronic supplementary material

Below is the link to the electronic supplementary material.

Supplementary Material 1

## Data Availability

No datasets were generated or analysed during the current study.

## Abbreviations

AD: Alzheimer disease
AKI: Acute kidney injury
ALD: Alcoholic liver disease
ALI: Acute lung injury
ARDS: Adult respiratory distress syndrome
COPD: Chronic obstructive pulmonary disease
CRP: C-reactive protein
CS: Cytokine Storm
DEGs: Differentially expressed genes
DIC: Disseminated intravascular coagulation
KEGG: Kyoto Encyclopedia of Genes and Genomes
MODS: Multiple organ dysfunction syndrome
MOF: Multiple organ failure
PCT: Procalcitonin
SOFA: Sepsis related organ failure assessment
STRING: Search Tool for the Retrieval Interacting Genes
T2DM: Type 2 diabetes mellitus
Th: T helper
Tregs: T regulatory
